# Measuring positive memories of home and family during childhood: The development and initial validation of the ‘Memories of Home and Family Scale’

**DOI:** 10.1007/s12144-022-03220-w

**Published:** 2022-06-17

**Authors:** Mark Shevlin, Enya Redican, Eoin McElroy, Menachem Ben-Ezra, Thanos Karatzias, Philip Hyland

**Affiliations:** 1 School of Psychology, Ulster University, Coleraine, UK; 2 Ariel University, Ariel, Israel; 3 School of Health and Social Care, Edinburgh Napier University, Edinburgh, UK; 4 Department of Psychology, Maynooth University, Maynooth, Ireland

**Keywords:** Benevolent childhood experiences, Adverse childhood experiences, Resilience

## Abstract

There is a burgeoning evidence base highlighting the positive influence of benevolent childhood experiences (BCEs), even in the context of adversity. However, few measures are available to assess BCEs. The current study sought to develop and validate a measure which assesses positive recollections of experiences and emotions at home and with family during childhood called the ‘Memories of Home and Family Scale’(MHFS). Confirmatory factor analysis (CFA) was employed to test the latent structure of the preliminary MHFS item scores in a sample of university students from the United Kingdom (*N* = 624). Following selection of the best-fitting model and final items for inclusion in the scale, total and subscale scores were correlated with a range of mental health outcomes. CFA results indicated that the latent structure of the MHFS items was best represented by a correlated six-factor first-order model*.* The final MHFS demonstrated high levels of internal reliability and convergent validity.

Adverse childhood experiences (ACEs), including abuse, maltreatment, and household dysfunction, are highly prevalent. Approximately 42.2% of adults in Europe and 60% of adults in North America have reported experiencing ACEs (Bellis et al., 2019) and these rarely occur in isolation (Kessler et al., 2010). Multiple ACE exposures are linked to pervasive and long-lasting physiological, psychological, and behavioral problems (Hughes et al., 2017; Petruccelli et al., 2019; Nelson et al., 2020). ACEs are estimated to account for three million disability adjusted life years (DALYs) per annum (Bellis et al., 2019) and 29.8% of all mental disorders globally (Kessler et al., 2010). Emerging evidence also indicates that the effects of ACEs can transmit across multiple generations (Narayan et al., 2021). Despite the negative health trajectories frequently linked to ACEs, not all individuals who experience ACEs during early development are negatively impacted (Danese & Lewis, 2021). Such individuals are considered as possessing high levels of resilience, described as an ability for positive adaption in the face of adversity (Rutter, 2006). Despite this, a substantial proportion of research has assumed a “deficit-based” approach emphasizing both risk and individual deficits associated with ACE exposures, whereas such research should be extended to develop an in-depth understanding of resilience enhancing factors which protect against the harmful effects of ACEs (Bartlett, 2020; McEwen & Gregerson, 2019).

Resilience, however, emphasizes the role of positive contextual, social, and individual factors in promoting positive adaption in the face of adversity (Zimmerman, 2013). Such factors are considered as being either assets or resources, the former describing internal resilience enhancing factors (e.g., coping skills) and the latter describing sources external to the individual (e.g., parental support) (Fergus & Zimmerman, 2005). In recent years, there has been growing interest in how benevolent childhood experiences (BCEs) cultivates resilience in young people. BCEs describe experiences during early development which foster a sense of safety, security, and predictability (Narayan et al., 2018). Research has shown how individuals with chronic ACE exposure who also report high levels of BCEs experience fewer PTSD (posttraumatic stress disorder) symptoms and stressful life events than those who have experienced multiple ACEs and few BCEs (Narayan et al., 2020). Similarly, other studies have demonstrated how many of the associations between ACEs and health outcomes attenuate or disappear after accounting for BCEs, suggesting that BCEs can counteract the deleterious effects of ACEs (Crandall et al., 2019, 2020; Kuhar & Zager Kocjan, 2021). Moreover, parental BCEs offer protection from the intergenerational transmission of ACEs (Narayan et al., 2021). Not only are BCEs beneficial in the presence of ACEs, BCEs have also been shown to positively influence mental health and wellbeing independent of ACEs (e.g., Doom et al., 2021). Overall, empirical evidence indicates that BCEs promote psychological wellbeing and healthy development and offer protection against the harmful effects of ACEs.

The Benevolent Childhood Experiences scale (BCE scale; Narayan et al., 2015, 2018) is the most frequently used measure of BCEs. Ten positive childhood experiences are included in the scale, and these center around themes of safety (e.g., ‘at least one caregiver with whom you felt safe’), support (e.g., ‘at least one good friend’,) and predictability (e.g., ‘predictable home routine’) (Narayan et al., 2018). The BCE scale uses a dichotomous response format to indicate the presence or absence of BCEs during the first eighteen years of life. A total summed score reflecting quantity of BCEs endorsed can be computed. The psychometric properties of the BCE scale have been supported across multiple samples including low-income pregnant women (Narayan et al., 2018), homeless parents (Merrick et al., 2019), treatment-seeking trauma-exposed adults (Karatzias et al., 2020) and other community adult samples (e.g., Almeida et al., 2021; Oge et al., 2020; Zhan et al., 2021). Other BCE measures include the Positive Childhood Experiences Score (Bethell et al., 2019) and the Childhood Caregiving Environment scale (CCE scale; Abbott & Slack, 2021). The former uses a dichotomous response format to assess frequency of exposure to seven BCEs related to family, peers, school, and the community while the latter utilizes an ordered multi-category response format to assess frequency of exposure to eight BCEs occurring within the family caregiving context. Although current measures are highly valuable, there remains some gaps which necessitate an additional BCE measure.

The BCE scale was designed as a counterpart of the widely utilized 10-item ACE questionnaire developed by Felitti et al. (1998) and contains the same number of items and response format as the original ACE questionnaire. However, the dichotomous response categories included in the ACE questionnaire fails to capture the frequency, intensity, or chronicity of exposures to adverse events (Anda et al., 2020) and this also applies to the BCE scale. For instance, Crouch et al. (2019) used a dichotomous response option representing frequency of exposure to BCEs (i.e., ‘some to most of the time’ or ‘little of the time to never’) and found that the presence of a stable, safe, and nurturing caregiver only reduced likelihood of distress for those who reported such experiences ‘some to most of the time’. These findings illustrated how frequency of such BCEs is an important factor in determining subsequent risk, and thus, an ordered multi-category response scale is likely to be advantageous.

The BCE scale includes objective BCEs in an effort to optimize likelihood of reliable retrospective reporting (Narayan et al., 2018). However, recollection of subjective emotions and behaviors during early years has been shown to be just as, if not more, influential in determining later outcomes compared to objective behaviors. Gilbert et al. (2003) demonstrated how childhood feelings of submissiveness were a primary predictor of later depression while recall of parental behavior was not. Such observations led to the development of the Early Memories of Warmth and Safeness Scale (EMWSS; Richter et al., 2009), a measure designed to assess recollections of personal emotions, feelings and experiences during early development (e.g., ‘I had a sense of belonging’, ‘I felt that I was a cherished member of my family’). Using this scale, Richter et al. (2009) found that subjective recall of positive feelings, emotions and experiences during childhood was a stronger predictor of depression, anxiety, stress, self-criticism, and self-reassurance in adulthood than recall of objective experiences. Similarly, adolescents who recalled more positive emotional memories from childhood experienced lower levels of depressive, anxiety, and stress symptomology (Cunha et al., 2014).

To align with developmental psychopathology perspectives as well as ecological systems and ecological-transactional perspectives (Bronfenbrenner, 1979; Cicchetti & Lynch, 1993), the BCE scale includes resources and experiences across multiple levels of a young person’s proximal environment including family, school, peers, and the community (Narayan et al., 2018; Narayan et al., 2020). However, there are no dedicated measures other than the CCE scale (Abbott & Slack, 2021) that measure BCEs specifically related to experiences within one’s family and home life. Assessing BCEs that occur within the familial and home environment is important given that the provision of loving and supportive caregiving equips a young person with the necessary skillsets to effectively negotiate later stress and adversity (Feder et al., 2019). Familial factors that have been frequently identified as enhancing resilience in the context of adversity include stable and supportive caregiving (Afifi & MacMillan, 2011), family cohesion, parental involvement, positive family climate, and positive parenting (Fritz et al., 2018). Moreover, close family relationships reduce internalizing and externalizing symptomology in individuals exposed to high levels of community violence (Ozer et al., 2017), while maternal and sibling warmth and a positive home atmosphere improve emotional and behavioral adjustment in young victims of bullying (Bowes et al., 2010). Thus, current research strongly indicates that there are many facets of family-related BCEs which can promote positive outcomes and offer protection from the negative effects of ACEs. However, it is unlikely that the eight-item CCE scale captures the full spectrum of BCEs that can occur within the family and home environment, and thus, an additional measure is warranted. Moreover, there are likely other BCEs which can be fostered within the familial environment. For instance, perceptions of belonging, belief that life has meaning, and emotional security have been implicated as resilience enhancing factors (Masten & Barnes, 2018; Masten, 2014; Masten, 2007), aspects not addressed in current BCE measures.

The family is considered a primary social group (Arce, 1970), and thus social comparison within the family may play a key role in determining quality of childhood experiences. Allan and Gilbert (1995) identified three core dimensions of social comparison including group fit (i.e., acceptance, similarity to other group members, belonging), attractiveness (i.e., likeability, attractiveness) and social rank (i.e., perceived inferiority, incompetence, weakness). Research has shown how perceived parental rejection (i.e., group fit) increases risk of internalizing and externalizing difficulties as well as poorer academic performance and prosocial behaviours in adolescents (Putnick et al., 2015). Recollections of feeling inferior and less favored in a family (i.e., social rank) have been linked to maladaptive psychopathological outcomes in young people (Gilbert & Gerlsma, 1999), while the familial environment plays a key role in increasing a young person’s sense of belonging (King & Boyd, 2016). Thus, BCEs within the familial environment which promote positive perceptions of group fit and social rank may be an important phenomenon to capture.

Consequently, there are some gaps which warrants the need for an additional measure. Therefore, the current study sought to develop a multidimensional measure which captures frequency of exposure to a wide spectrum of subjective memories of positive experiences and emotions which can occur within the family and home environment. Similar to how the BCE scale was developed as a counterpart to the ACE questionnaire, our measure was intended to act as a positive analogue to a frequently employed multidimensional measure of childhood maltreatment - the short-form of the Childhood Trauma Questionnaire (CTQ-SF; Bernstein et al., 2003). Existing measures including BCE measures, the EMWSS scale (Richter et al., 2009) and Social Comparison Scale (Allan & Gilbert, 1995) were drawn upon to develop a comprehensive list of BCEs. We sought to test the performance of these items to develop a finalized set of items which would form part of the final scale entitled the Memories of Home and Family scale (MHFS). Following selection of the finalized items, it was hypothesized that higher MHFS total and sub-scale scores would be associated with lower scores on measures of depression, anxiety, stress and loneliness (Bethell et al., 2019; Crandall et al., 2019; Narayan et al., 2020; Narayan et al., 2018; Doom et al., 2021; Wang et al., 2021) and higher scores on a measure of resilience (Kocatürk & Çiçek, 2021).

## Methods

### Participants

Participants were 624 university students registered at two universities in the UK. Participants were recruited using opportunity sampling from undergraduate and postgraduate Psychology degree programs; emails were distributed to all students and a link was provided to the survey hosted by Qualtrics. Ethical approval was granted by the Ethical Boards at each institution. The mean age of the sample was 24.33 (SD = 10.75, range = 10–78 years). Most participants were female (76.4%, *n* = 477), had no children (87.7%, *n* = 73) and identified as white (77.5%, *n* = 482). Nearly a third of the sample reported being in a committed relationship (57.9%, *n* = 361).

### Measures

#### Memories of Home and Family

A preliminary 31-item version of the memories of home and family scale (MHFS) was developed. Items were generated under 6 domains, and these domains emerged from reviewing existing measures related to benevolence and positive experiences in childhood. An initial item pool for each domain were initially generated by 2 members of the research team (MS & PH) and these were reviewed, revised and amended by the other members of the team. The domains were (1) Being a valued member of family (e.g., ‘I felt my parents valued me’ and ‘I felt that I was an important part of my family’*)* (2) Being an independent member of the family (e.g., ‘I contributed to family discussions and decisions’, ‘I thought of myself as equal to other family members), (3) Feeling supported within family (e.g., ‘My family were supportive’, ‘My parents gave me freedom to be myself’*)*, (4) Feeling secure within family (e.g., ‘I felt secure at home’, ‘I slept well at home’), (5) Feeling a sense of wellbeing at home (e.g., ‘I was happy at home’, ‘If times were tough my family helped me feel better’), and (6) Experiences of growth and meaning (e.g., ‘My home-life allowed me to feel my life was meaningful’, ‘At home I had a sense of purpose’*)*.

The following instructions were provided to participants: “The following questions are designed to explore your memories of your childhood at home and with your family. The following questions are about how you recall your early life up to the age of 16 years. Please complete the scale by circling the most appropriate number under each statement”. Participants were asked to indicate the frequency to which they experienced each of the items using a five-point Likert scale ranging from ‘Never’ (1) to ‘Always’ (5).

#### Depression, Anxiety, and Stress Symptomology

The short form version of the depression, anxiety, and stress scale (DASS-21; Henry & Crawford, 2005) was used to assess depression, anxiety, and stress symptomology. Respondents are asked to indicate the extent to which each statement applied to them over the past week using a four-point Likert scale ranging from ‘did not apply to me at all’ (0) to ‘applied to me very much or most of the time’ (3). Total DASS-21 scores were computed by summing responses to all items. Sub-scale scores were computed by summing responses to all seven-items within each subscale. Higher total and sub-scale scores indicated higher symptom severity. Cronbach’s alpha for total DASS-21 score (α = .95) and for the stress (α = .87), anxiety (α = .84) and depression (α = .91) in the current study were excellent.

#### Resilience

The Brief Resilience Scale (BRS; Smith et al., 2008) was used to assess resilience. The BRS is a six-item scale and includes items such as ‘I tend to bounce back quickly after hard times’ and ‘it does not take me long to recover from a stressful event’. Respondents are asked to indicate their level of agreement with each statement using a five-point Likert scale ranging from ‘strongly disagree’ (1) to ‘strongly agree’ (5). A total resilience score is derived from summing responses to all items, and higher scores indicate higher levels of resilience. Cronbach’s alpha for the BRS in the present study was excellent (α = .86).

#### Loneliness

The short-form De Jong Gierveld Loneliness scale (DJGLS; Gierveld & Tilburg, 2006) was used to measure loneliness. The DJGLS is a six-item scale and consists of items including ‘I experience a general sense of emptiness’ and ‘I miss having people around’. Respondents are asked to indicate the frequency of each statement using a five-point Likert scale ranging from ‘none of the time’ (1) to ‘all of the time’ (5). A total loneliness score is derived from summing responses to all items, and higher scores indicate higher levels of loneliness. Cronbach’s alpha for the DJGLS in the current study was satisfactory (α = .74).

### Analytic Procedure

Confirmatory factor analysis (CFA) was employed to test the latent structure of the MHFS item scores. Three alternative models were tested; Model 1 was a unidimensional CFA model where all MHFS items loaded on to a single latent variable (positive memories), Model 2 was a correlated six-factor first-order CFA model where all MHFS items loaded onto their respective first-order latent variables (i.e., valued, independence, support, security, wellness, growth and meaning), and Model 3 was a second-order CFA model where all first-order latent variables (i.e., valued, independence, support, security, wellness, growth and meaning) loaded onto a single latent variable (positive memories).

The model was tested using Mplus 8.0 (Muthén & Muthén, 2017) with robust maximum likelihood estimation (Yuan & Bentler, 2000). Goodness of fit was assessed using the chi-square statistic, the comparative fit index (CFI; Bentler, 1990), the Tucker-Lewis index (TLI; Tucker & Lewis, 1973), the root mean square error of approximation (RMSEA; Browne & Cudeck, 1992; Steiger, 1990) and the Standardized Root Mean Square Residual (SRMR; Jöreskog & Sörbom, 1981). Using standard cut-off criteria (Hu & Bentler, 1999), a non-significant chi-square (p > .05) indicated good fit; CFI and TLI values ≥ .90 considered adequate and ≥ .95 considered excellent model fit, respectively; SRMR values ≤0.8 indicating good fit; RMSEA values <.05 indicating close fit and < .08 indicating adequate fit (Steiger, 1990). Bayesian Information Criterion (BIC; Sclove, 1987), sample size adjusted BIC (ssaBIC; Sclove, 1987) and Akaike Information Criterion (AIC; Akaike, 1987) were used to compare fit of competing models with lower values indicative of superior model fit. A 10-point difference in BIC values indicated that the lower BIC model should be selected (Raftery, 1995).

Following selection of the best-fitting model and final items for inclusion in the MHFS, total scale and sub-scale scores were correlated with total and sub-scale DASS-21 scores, and total scores on the BRS and DJGLS*.* Correlations were calculated using SPSS Version 27.

## Results

Model fit results are presented in Table 1. Model 1 was rejected due to poor model fit while Model 2 and Model 3 had good fit. Chi-squared statistic was significant for both Model 2 and Model 3, however this should not be considered as evidence for rejection of these model as the chi-squared statistic is highly sensitive to sample size (Tanaka, 1987). Compared to Model 3, Model 2 had the lowest BIC, the lowest RMSEA as well as the highest TLI and CFI values. Thus, Model 2 was selected as the best-fitting model. All items loaded significantly and strongly onto their respective first-order factors with the exception of one reverse-coded item *“I often had to do things others in my family wanted – even when I did not want to”* which had a weak (.03) and non-significant loading (p > .05). Two other items *“My parents controlled me by using threats and punishments”* and “*Being at home was relaxing”* loaded weakly (<.40), but significantly (p < .001) onto their respective factors. Consequently, these three items were not considered for the final scale. The exclusion of these three-items resulted in improved fit of the correlated six-factor model (*ΔTLI* = .024, *ΔCFI* = .024, *ΔRMSEA* = .004, *ΔSRMR* = .005) . All items loaded moderately to strongly (.43–.93) and significantly (p < .001) onto their respective first-order factors (see Table 2) and correlations among all first-order factors ranged from .68 to .92 (see Table 3). Thus, the final MHFS is a 28-item scale which provides both an overall MHFS score and/or individual sub-scale scores.

**Table 1:** Fit Statistics for the Confirmatory Factor Analysis of the Memories of Home and Family Scale

CFA Model	χ^2^ (df) p	TLI	CFI	RMSEA (95% C.I.)	SRMR	BIC
Original 31-items						
Unidimensional	2766.136 (434), p < .001	0.782	0.797	0.093 (0.090, 0.096)	0.063	44366.366
6-factor first-order	1097.680 (419), p < .001	0.934	0.941	0.051 (0.047, 0.055)	0.039	42223.189
Second Order	1370.957 (428), p < .001	0.911	0.918	0.059 (0.056, 0.063)	0.053	42517.596
Final 28-item scale						
Unidimensional	2443.933 (350), p < .001	0.788	0.804	0.098 (0.094, 0.102)	0.064	38711.523
6-factor first order	871.872 (335), p < 0.001	0.950	0.943	0.051 (0.047, 0.055)	0.034	36590.962
Second order	959.022 (336), p < 0.001	0.934	0.942	0.055 (0.051, 0.059)	0.083	36702.575

*χ*^*2*^ chi-square test, *TLI* Tucker Lewis Index, *CFI* Comparative Fit Index, *RMSEA* Root Mean Square Error of Approximation, Standardized Root Mean Square Residual, *BIC* Bayesian Information Criterion

**Table 2:** Standardized Factor Loadings from the Correlated 6-factor Model of the MHFS

Item	Valued	Indep	Support	Secure	Well	Growth
1. I felt I could be myself around my family	.764**					
2. I felt my parents valued me	.892**					
3. I felt that I was an important part of my family	.899**					
4. I felt appreciated by my family	.930**					
5. I felt like I made a valuable contribution to my family life	.775**					
6. I thought of myself as equal to other members of my family		.819**				
7. My family listened to me		.886**				
8. I contributed to family discussions and decisions		.431**				
9. I felt my opinions were heard by my family		.891**				
10. My family were supportive			.812**			
11. My parents praised me			.733**			
12. My parents gave me freedom to be myself			.874**			
13. The atmosphere at home was encouraging and supportive			.845**			
14. When I needed them, I could rely on my family			.886**			
15. I felt secure at home				.897***		
16. My home was a safe place for me to be				.798***		
17. A lot of the time I didn’t want to be at home				.579**		
18. I slept well at home				.709**		
19. I knew my parents were looking out for me				.852**		
20. I felt anxious or nervous at home					.612**	
21. I was happy at home					.841**	
22. If times were tough my family helped me feel better					.879**	
23. I worried being around my family					.592**	
24. I could develop and grow as a person at home						.838**
25. My home-life allowed me to feel my life was meaningful						.917**
26. At home I felt I had a sense of purpose						.866**
27. My family supported me in reaching my goals						.852**
28. I felt encouraged to take charge of my life						.795**

**Table 3:** Factor Correlations from the CFA of the MHFS Domains

	1. Valued	2. Independence	3. Support	4. Secure	5. Wellness
1	–				
2	.896	–			
3	.921	.894	–		
4	.877	.801	.930	–	
5	.714	.684	.765	.799	–
6	.725	.910	.780	.736	.910

All correlations are significant at *p* < .001

Item level statistics for the 28 items as well as information on skew and kurtosis are reported in Table 4. The range of possible scores for the new scale was 28 to 140 and the observed range of scores was 42 to 140, with a mean score of 114.24 (SD = 21.79). Composite reliability (CR) estimates were excellent for the total MHFS and for each of the sub-scales (overall = .98, valued = .93, independence = .86, support = .92, secure = .88, wellness = .83, growth and meaning = .93). The bivariate correlations of MHFS total scores and the DASS-21 (total and subscale), loneliness total score and resilience total score were calculated. The total MHFS scores were significantly and negatively correlated with the DASS-21 total (r = −.34, p < .001) and the Depression (r = −.35, p < .001), Anxiety (r = −.27, p < .001) and Stress (r = −.30, p < .001) subscale scores. It was also negatively correlated with the scores from the loneliness measure (r = −.53, p < .001) and positively associated with the scores from the resilience measure (r = .26, p < .001). Associations between the MHFS sub-scale scores and the mental health outcomes were largely similar to those observed for the MHFS total score (see Table 5).

**Table 4:** Item-level Descriptive Statistics for the 28-item Positive Memories of Childhood and Family Scale

	Mean	SD	Range	Item-total correlation	Skew (SE)	Kurtosis (SE)
1. I felt I could be myself around my family	4.18	0.99	1–5	0.775**	−1.040 (0.098)	0.368 (0.196)
2. I felt my parents valued me	4.31	0.95	1–5	0.828**	−1.184 (0.098)	0.367 (0.196)
3. I felt that I was an important part of my family	4.25	0.96	1–5	0.827**	−1.108 (0.098)	−0.923 (0.098)
4. I felt appreciated by my family	4.18	0.99	1–5	0.855**	−0.923 (0.098)	−0.172 (0.196)
5. I felt like I made a valuable contribution to my family life	3.95	1.06	1–5	0.739**	−0.682 (0.098)	−0.526 (0.196)
6. I thought of myself as equal to other members of my family	3.85	1.15	1–5	0.770**	−0.748 (0.098)	−0.277 (0.196)
7. My family listened to me	3.88	1.07	1–5	0.812**	−0.682 (0.098)	−0.526 (0.196)
8. I contributed to family discussions and decisions	3.69	1.17	1–5	0.437**	−0.431 (0.098)	−0.870 (0.196)
9. I felt my opinions were heard by my family	3.77	1.14	1–5	0.793**	−0.574 (0.098)	−0.654 (0.196)
10. My family were supportive	4.24	0.96	1–5	0.857**	−1.067 (0.098)	0.273 (0.196)
11. My parents praised me	4.10	1.01	1–5	0.777**	−0.815 (0.098)	−0.323 (0.196)
12. My parents gave me freedom to be myself	4.02	1.07	1–5	0.741**	−0.873 (0.098)	−0.155 (0.196)
13. The atmosphere at home was encouraging and supportive	3.97	1.02	1–5	0.843**	−0.640 (0.098)	−0.468 (0.196)
14. When I needed them, I could rely on my family	4.26	0.98	1–5	0.804**	−1.172 (0.098)	0.591 (0.196)
15. I felt secure at home	4.32	0.99	1–5	0.834**	−1.283 (0.098)	0.558 (0.196)
16. My home was a safe place for me to be	4.37	0.98	1–5	0.748**	−1.521 (0.098)	1.561 (0.196)
17. A lot of the time I didn’t want to be at home (reverse)	3.76	1.26	1–5	0.587**	−0.734 (0.098)	−0.554 (0.296)
18. I slept well at home	4.29	0.94	1–5	0.679**	−1.186 (0.098)	0.524 (0.196)
19. I knew my parents were looking out for me	4.41	0.87	1–5	0.816**	−1.308 (0.098)	0.835 (0.196)
20. I felt anxious or nervous at home (reverse)	3.86	1.20	1–5	0.552**	−0.813 (0.098)	−0.344 (0.196)
21. I was happy at home	4.10	0.98	1–5	0.773**	−0.889 (0.098)	0.095 (0.196)
22. If times were tough my family helped me feel better	4.00	1.10	1–5	0.791**	−0.857 (0.098)	−0.172 (0.196)
23. I worried being around my family	4.13	1.10	1–5	0.541**	1.155 (0.098)	0.494 (0.196)
24. I could develop and grow as a person at home	4.01	1.05	1–5	0.762**	−0.803 (0.098)	−0.287(0.196)
25. My home-life allowed me to feel my life was meaningful	3.94	1.13	1–5	0.806**	−0.835 (0.098)	−0.257 (0.196)
26. At home I felt I had a sense of purpose	3.80	1.14	1–5	0.743**	−0.575 (0.098)	−0.707 (0.196)
27. My family supported me in reaching my goals	4.18	1.01	1–5	0.775**	−1.011 (0.098)	0.028 (0.196)
28. I felt encouraged to take charge of my life	4.24	0.96	1–5	0.728**	−0.951 (0.098)	−0.041 (0.196)

**Table 5:** Bivariate correlations for MHFDC total and sub-scale scores and mental health outcomes

	DASS-21 Total score	DASS-21 Depression	DASS-21 Anxiety	DASS-21 Stress	Loneliness	Resilience
Total	−.34**	−.35**	−.27**	−.30**	−.53**	.26**
Valued	−.35**	−.35**	−.29**	−.30**	−.48**	.24**
Independence	−.33**	−.34**	−.26**	−.30**	−.45**	.25**
Support	−.29**	−.29**	−.23**	−.24**	−.48**	.24**
Secure	−.35**	−.33**	−.29**	−.30**	−.45**	.21**
Wellness	−.30**	−.29**	−.25**	−.29**	−.42**	.19**
Growth & Meaning	−.25**	−.27**	−.18**	−.22**	−.46**	.20**

## Discussion

The current study sought to develop and validate a multidimensional measure to assess recollections of positive emotions and experiences at home and with family during childhood, called the Memories of Home and Family Scale (MHFS). Aligning with our original objective to develop a scale which captures multiple dimensions of memories of home and family during childhood, CFA results strongly supported the representation of the MHFS as a multidimensional measure. Consequently, the final MHFS is a 28-item measure which can be considered as a positive analogue to the 28-item CTQ-SF (Bernstein et al., 2003), and includes items pertaining to memories of feeling like a valued family member, an independent family member, supported within the family, secure within the family, having a sense of wellbeing at home and having opportunities for growth and meaning during childhood.

Results illustrated that the internal reliability of the overall MHFS and sub-scales were high (i.e., >.80) while the total MHFS scores correlated with criterion variables in the predicted direction; negatively with depression (r = −.35), anxiety (r = −.27), stress (r = −.30) and loneliness (r = −.53) and positively with resilience (r = .26). The magnitude of the correlations were somewhat similar to those reported by Doom et al. (2021) based on a survey of US students during Covid-19 pandemic: depression (r = −.35), anxiety (r = −.30), stress (r = −.35), and loneliness (r = −.29). This indicates that the MHFS is likely measuring a conceptually similar dimension to the BCE scale, and that the subjective aspect of the MHFS may have resulted in the strong association with loneliness, which is an evaluative construct. This may also represent a mechanism where early positive experiences engender confidence and skills in social interactions.

The MHFS adds to current measures of BCEs with respect to its’ structure and content. In terms of structure, the inclusion of ordered multiple response categories strengthens the sensitivity and reliability of the MHFS (Cheng et al., 2012), while the ability to explore the frequency of positive childhood memories related to the family and home environment may assist in deciphering the characteristics of BCEs which increase potential for positive outcomes in the face of adversity. Similarly, the ability to calculate total and sub-scale scores may prove beneficial in isolating the effects of different facets of BCEs on psychological wellbeing and health. In terms of content, the MHFS adds to a small repertoire of measures which assess subjective memories of positive emotions and experiences during childhood such as the Early Life Experiences Scale which assesses memories of feeling devalued, frightened and submissive during early years (Gilbert et al., 2003) and the EMWSS (Richter et al., 2009). Given that positive memories of subjective emotions during childhood have been shown to predict more favourable outcomes (e.g., Cunha et al., 2012; Gilbert et al., 2003; Richter et al., 2009), it will be of interest to determine the role of positive memories of home and family during childhood in predicting later outcomes. The inclusion of a vast array of BCEs is also a notable distinguishing characteristic of the scale. For instance, the inclusion of items pertaining to opportunities for growth and meaning may prove insightful given that meaning-making is considered a fundamental aspect of increasing resilience during difficult experiences (Liebenberg, 2020).

Results from the current study should be considered in light of several limitations. The use of a university-based sample limits generalisability of findings to general-population and clinical samples. Moreover, our results may be somewhat limited by the relatively small size of the sample, over-representation of females and the sampling strategy employed. Therefore, replication of findings is required in larger representative samples. Similar to Narayan et al. (2018), almost a quarter of participants (60.6%; *n* = 378) endorsed all twenty-eight BCEs as occurring ‘frequently’ or ‘always’ during childhood, indicating potential ceiling effects. Such high endorsement of positive memories of home and family is unlikely to translate to highly burdened samples, and thus establishing how the MHFS holds in treatment-seeking samples is paramount. Similarly, the MHFS latent factors were highly-correlated in the current study and thus individual sub-scale scores may not hold significant predictive utility or validity. This may be a characteristic of the university population investigated in the current study, especially given that average MHFS total score was within the upper range of potential scores. Given that the purpose of this study was to develop and provide initial validation for the MHFS, more detailed investigations of the validity and reliability of the MHFS is necessary.

Nevertheless, given the unique strengths of the MHFS, it is anticipated that this measure will be routinely employed by researchers and clinicians alike. Important lines for future enquiry include detailed investigations of the validity, reliability, and predictive utility of the MHFS across diverse samples. It would be of interest to include the MHFS in research using both the BCE scale and the ACE questionnaire. This is for two reasons; (1) to determine whether the MHFS is tapping into a different domain of BCEs than the BCE scale and whether positive memories of home and family offer additional protective effects compared to recollection of objective experiences as included in the BCE scale and (2) to determine whether BCEs as measured by the MHFS represent a distinct albeit related construct from ACEs. To facilitate such research, it may be beneficial to replicate the methodological procedure adopted by Karatzias et al. (2020) who utilised CFA to determine whether BCEs as measured by the BCE scale and ACEs represented distinct albeit related constructs or opposing ends of a continuum of childhood experiences, with findings from this study supporting the former. Should positive experiences of home and family help mitigate the effects of ACEs, evidence-based programs and policies which emphasise improving potential for BCEs within the familial context may be especially important. For instance, the Strengthening Families programme (Kumpfer & DeMarsh, 1985; Kumpfer et al., 2010; Kumpfer & Magalhães, 2018) is a family-centred approach which focuses on increasing family protective factors and reducing risk factors within the familial context. Actively screening for the presence of BCEs may prove to be a helpful mechanism to identify at-risk individuals (Merrick & Narayan, 2020), while interventions which draw on memories of BCEs may serve as a resource in increasing recovery potential in traumatised individuals (Karatzias et al., 2020).

## Data Availability

Due to the nature of this research, participants of this study did not agree for their data to be shared publicly, and therefore, supporting data is not available.
